# Integrated Computational Approaches for Drug Design Targeting Cruzipain

**DOI:** 10.3390/ijms25073747

**Published:** 2024-03-27

**Authors:** Aiman Parvez, Jeong-Sang Lee, Waleed Alam, Hilal Tayara, Kil To Chong

**Affiliations:** 1 Department of Electronics and Information Engineering, Jeonbuk National University, Jeonju 54896, Republic of Korea; aimanparvez@jbnu.ac.kr (A.P.); waleedtkr@jbnu.ac.kr (W.A.); 2 Department of Functional Food and Biotechnology, College of Medical Sciences, Jeonju University, Jeonju 55069, Republic of Korea; jslee11@jj.ac.kr; 3 School of International Engineering and Science, Jeonbuk National University, Jeonju 54896, Republic of Korea; 4 Advances Electronics and Information Research Center, Jeonbuk National University, Jeonju 54896, Republic of Korea

**Keywords:** deep learning model, pharmacophore model, QSAR, molecular docking, MD simulation

## Abstract

Cruzipain inhibitors are required after medications to treat Chagas disease because of the need for safer, more effective treatments. *Trypanosoma cruzi* is the source of cruzipain, a crucial cysteine protease that has driven interest in using computational methods to create more effective inhibitors. We employed a 3D-QSAR model, using a dataset of 36 known inhibitors, and a pharmacophore model to identify potential inhibitors for cruzipain. We also built a deep learning model using the Deep purpose library, trained on 204 active compounds, and validated it with a specific test set. During a comprehensive screening of the Drug Bank database of 8533 molecules, pharmacophore and deep learning models identified 1012 and 340 drug-like molecules, respectively. These molecules were further evaluated through molecular docking, followed by induced-fit docking. Ultimately, molecular dynamics simulation was performed for the final potent inhibitors that exhibited strong binding interactions. These results present four novel cruzipain inhibitors that can inhibit the cruzipain protein of *T. cruzi*.

## 1. Introduction

**Figure 1:**
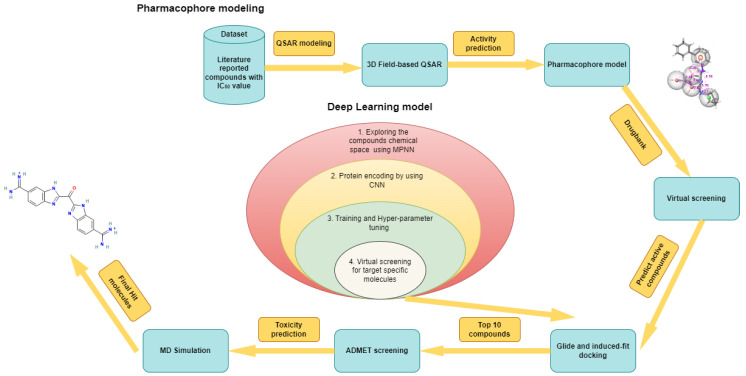
Schematic representation of 3D-QSAR modeling, pharmacophore generation, drug-like database designing, and virtual screening, which were performed using the Phase module of Schrodinger. Following that, the deep learning model was built using CNN and MPNN from the Deep purpose library [20]. Additionally, molecular docking and molecular dynamic simulation were carried out using Glide, induced-fit docking, and Desmond, respectively.

**Figure 2:**
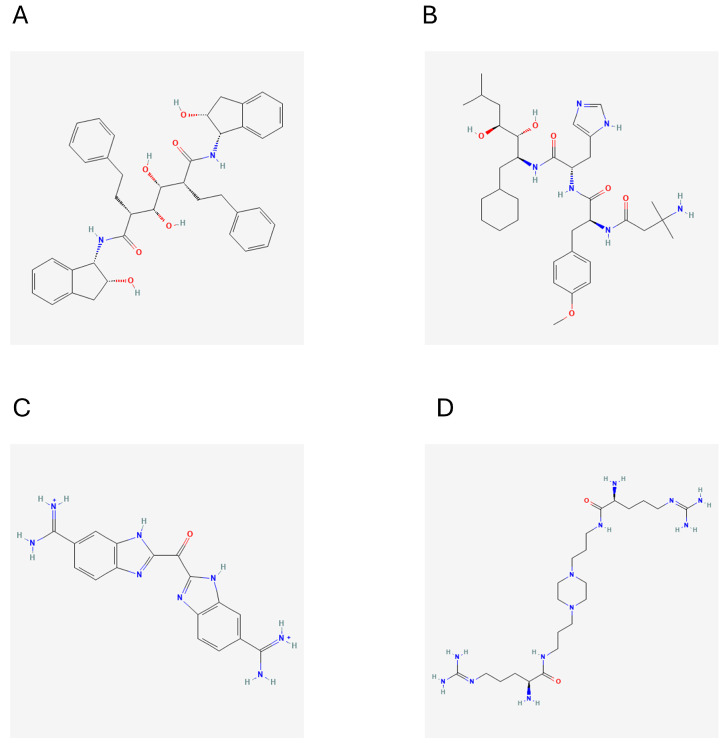
Shows the structural information of the 4 hit compounds (**A**) DB02704, (**B**) DB03395, (**C**) DB03213, and (**D**) DB15199.

*Trypanosoma cruzi* (*T. cruzi*), an intracellular protozoan parasite, serves as the etiological agent responsible for Chagas disease. Latin America has a significant public health issue due to 6–8 million untreated cases of this condition. Alarmingly, as it develops, there are still 30,000 new cases reported annually [1]. The only two licensed pharmacological treatments for Chagas disease are nifurtimox and benznidazole (BNZ). However, when subjected to lengthy treatment procedures, their effectiveness shows a decline [2,3]. Patients on therapeutic dosages of medications frequently have unfavorable side effects, which can sometimes lead to cardiac problems. As a result of these side effects, many patients discontinue their treatments due to discomfort [4]. Likewise, *T. cruzi* can cause a two-stage illness in different mammalian species, which spreads through congenital infections, blood transfusions, or the blood-feeding insects of the Reduviidae family [5]. The first stage, sometimes called the acute phase, is primarily asymptomatic and marked by increased parasitemia levels. The mortality rate during this stage is low, and any clinical symptoms that may be present typically disappear after eight weeks from the time of infection. During the second stage, often known as the chronic stage, serological testing can show decreasing quantities of parasites in the blood as well as the existence of *T. cruzi* antibodies. The sickness progresses slowly throughout this period, which can last anywhere from 10 to more than 30 years. About 30–40% of people eventually develop the disease’s distinctive symptoms. These symptoms may include abnormal radiography or electrocardiographic (ECG) test findings [5]. In addition, the *T. cruzi* causes three types of sickness: cardiac, digestive (which can cause megaesophagus and megacolon development), and cardio digestive. When a person’s immune system is weakened, as it is in AIDS patients, or when corticosteroids are used, the condition might progress more quickly in its chronic stage. In these situations, it is vital to carefully monitor and manage patients. This professional revision now describes the phases of Chagas disease more concisely and precisely [6]. The development of novel drugs whose therapeutical impact is based on activities as selective as possible on pathogen biomolecular targets is one of the primary techniques for developing safer and more effective medications for parasite disorders. The protozoan parasite *T. cruzi* has a variety of enzymes and molecular receptors that have been discovered and proposed as possible drug development targets [7]. One of the important molecular targets of significance is the enzyme cruzipain (CZP), which serves as the primary protease within the parasite’s physiological milieu [8]. The evidence supporting CZP as a target for therapeutic development is strong due to its inhibition of the enzyme causing parasite mortality [9]. However, the Chagas disease required a suitable and reliable drug that inhibits the effect of the cruzipain protein, helping in the complete recovery of the patient. Therefore, in silico work is required to identify the effective drug using the repurposing technique. In this study, we employed in silico methods and deep learning models to identify the potential inhibitors against the target protein cruzipain. These methods were used in a virtual screening to explore the Drug Bank database to identify novel cruzipain inhibitors. Meanwhile, it is worth noting that the Drug Bank maintains a large database of FDA-approved and investigational medicines, biotechnology compounds, and nutraceuticals [10,11]. A particularly beneficial undertaking in the field of drug discovery and development is running virtual screening (VS) programs targeted at drug repurposing, which entails investigating additional or subsequent therapeutic uses for well-established medicines. The utilization of VS to analyze chemical libraries that compile well-known therapeutics can be viewed as a type of knowledge-based rational drug repositioning [12,13,14,15,16] (based on chemo- and bioinformatics, among other things), which has recently been recognized as a useful tactic to help find new treatments for uncommon and untreated conditions [17,18,19]. The resulting compounds of VS are 1012 and 340 from pharmacophore and deep learning, respectively. These compounds were subsequently subjected to molecular docking analysis to evaluate their binding affinities with the target protein. To further assess the interacting stability of the protein with each compound, the top 10 candidates were selected for molecular dynamics simulations. After the simulations, we identified four promising hit compounds that exhibited the most favorable interactions and stability with the target protein. These compounds were identified as DB02704, DB03395, DB03213, and DB15199. The details of the work plan and 4 hit compounds are represented in the flow chart diagram, as shown in Figure 1 and Figure 2.

## 2. Results

### 2.1. Three-Dimensional Field-Based QSAR Model

**Figure 3:**
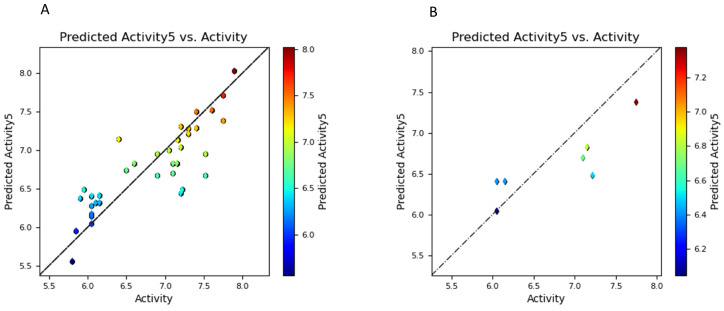
Scatterplot of the 3D QSAR (**A**) training set values; and (**B**) test set values.

Based on a Gaussian field methodology, a 3D Comparative Molecular Similarity Indices Analysis (QSAR) model was created (a CoMSIA-like model). To demonstrate connections between the electrostatic, hydrophobic, and steric fields of the 36 aligned chemicals and their known biological actions, this research project set out to build solid models. However, to reduce the overfitting of the model, the partial least squares (PLS) regression approach was used with a constraint of five components (as shown in Table 1). A training dataset of 27 compounds was carefully picked for the model generation procedure, while seven chemicals were simultaneously placed aside consciously to serve as a test dataset and utilized for model validation. A coefficient correlation ($r^{2}$) value of 0.73 (Figure 3) shows the strong predictive power of the derived models, indicating the best relationship between the structures and their activities. Moreover, this association underlines the model’s potential value in revealing the structure–activity correlations for the chemicals under study in addition to supporting the model’s prediction ability. The optimal (COMISA-like) model demonstrated a notable correlation coefficient value of $r^{2}$ = 0.685, indicating a substantial relationship between the structures and their activities. In addition, the model’s statistical significance in the regression process is supported by the F-value (F = 12.6) and the corresponding *p*-value (*p* = $5.98\times 10^{-6}$). The *p*-value, which is an essential indicator of the degree of significance associated with the F-test value, is exceptionally low in this instance, demonstrating our high level of confidence in our model. Additionally, we measured the root-mean-square error (RMSE = 0.40) and the regression’s standard deviation (SD = 0.375) to assess the overall model’s correctness, as shown in Table 1. These measures produced comparatively low values, indicating that there is little overall error present in both the model generation and prediction processes. The above statistical analyses allow us to conclude that our model is reliable and suitable for usage in further scenarios.

### 2.2. Pharmacophore Model

**Figure 4:**
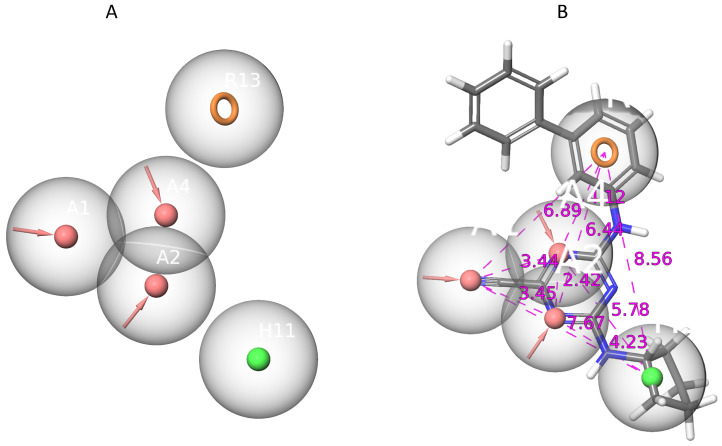
Chemical characterization of the selected pharmacophore. (**A**) the selected pharmacophore has a total of five features including three hydrogen acceptors (AAA), as well as one hydrophobic (H) and one aromatic ring (R). (**B**) The inner-features distance of the selected pharmacophore is displayed in angstrom (Å).

The QSAR dataset was utilized for ligand-based pharmacophore modeling, revealing consistent features such as hydrogen acceptors, hydrophobic elements, and aromatic rings, as shown in Figure 4. We generated 19 hypotheses to optimize our pharmacophore and match it with the chosen training set of compounds’ structural characteristics. However, the top five pharmacophore hypotheses were selected based on their phase-hypo score and are displayed in Table 2. Meanwhile, the leading hypothesis, AAAHR_1, has five pharmacophoric properties including three hydrogen acceptor sites (A), one hydrophobic area (H), and one aromatic ring (R). Notably, this hypothesis has a BEDROC score of 0.950, a volume score of 0.872, a vector score of 1.0, and a phase-hypo score of 1.27. In our pharmacophore modeling project, the existence of several functional groups, which provide crucial qualities like hydrogen bond acceptance, aromatic ring interaction, and hydrophobic character, offers a logical and reasonable outcomes.

### 2.3. Validation

The top-ranked pharmacophore hypothesis, AAAHR_1, was validated using a deep decoy set approach. This approach aims to assess the hypothesis’s ability to discriminate between a collection of active compounds and a collection of inactive decoys. The validation dataset consisted of 36 known active molecules from the literature and 1440 compounds with no recorded activity. The hit score, enrichment factor, and ROC are important indicators used to assess model performance. A higher hit score, ranging from 0 to 1, denotes better model quality. Surprisingly, the enrichment factor and ROC both received scores of 100.04 and 1.0. These findings make our pharmacophore theory acceptable for use in virtual screening applications and strongly imply its effectiveness.

### 2.4. Deep Learning Model

**Figure 5:**
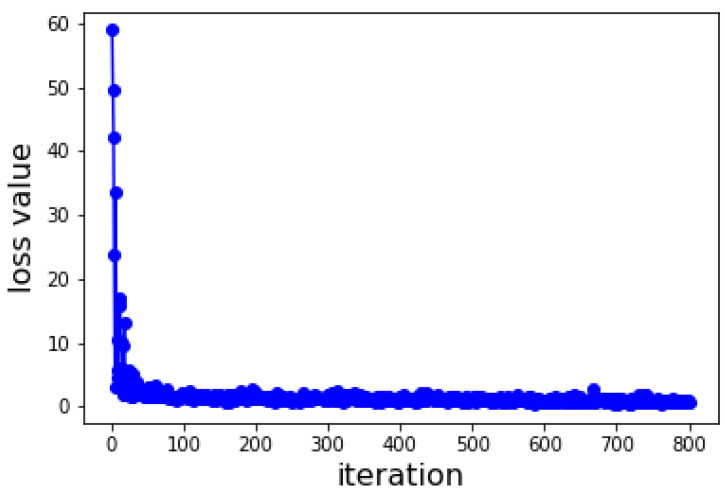
Loss function graph of the deep learning process.

The Deep Purpose framework was used to modify the deep learning model, which was trained on the prepared dataset. The model testing produced a mean square error (MSE) value of 0.62. Greater prediction accuracy is correlated with a lower MSE value. Additionally, the proposed model-computed Pearson correlation coefficient is 0.856, indicating a significant connection between the model variables. The strength of these correlations becomes more apparent when the Pearson correlation increases to a higher value. The concordance index (C-index) was used to evaluate the prediction performance in terms of survival times. The model’s C-index score of 0.826 indicated that it could predict survival periods with a higher degree of probability. Based on the loss function value, a graphical representation of the overall model performance is shown in Figure 5.

### 2.5. Virtual Screening

Both pharmacophore modeling and deep learning techniques were used to carry out the parallel virtual screening of the Drugbank database. Based on their phase-hypo scores, 1012 substances that demonstrated compatibility with the pharmacophore characteristics were chosen. Meanwhile, the top 340 compounds were simultaneously discovered by the deep learning-based screening method, which prioritized them based on their projected activity ratings. These findings were then considered for additional research using docking studies.

### 2.6. Molecular Docking Studies

#### 2.6.1. Glide SP (Standard Precision) Docking

In our docking studies, we utilized a total of 1352 compounds. Among them, 328 ligands were discovered via deep learning virtual screening, and 1012 were found using pharmacophore screening. The screened compounds were docked onto the T.cruzi cruzipain target protein using Glide SP docking, with the active sites of the T.cruzi cruzipain protein identified between residues GLN-19, CYS-25, GLY-65, GLY-66, LEU-67, ASP-161, HIS-162, ASN-182, TRP-184, and GLU-208. The top 10 ligand molecules from each dataset were chosen based on their Glide ratings. As shown in Table S3, ligands derived by the deep learning model have Glide scores ranging from −6.67 to −9.39 kcal/mol. The highest scoring molecule achieved a maximum Glide score of −9.39 kcal/mol. In addition, the compound with a higher binding affinity (−9.39) formed a hydrogen bond with GLU-117 and salt bridge bonds with ASP-161 and GLU-208. Alternatively, the ligands produced from the pharmacophore model showed Glide scores ranging from −8.056 to −10.36 kcal/mol are present in Table S4. Notably, the dataset yielded a ligand with a high Glide score of −10.36 kcal/mol, forming two hydrogen bonds with GLY-96 and ASP-18. Finally, from both pharmacophores and deep learning models, we obtained active compounds for further research.

#### 2.6.2. Induced Fit Docking

**Figure 6:**
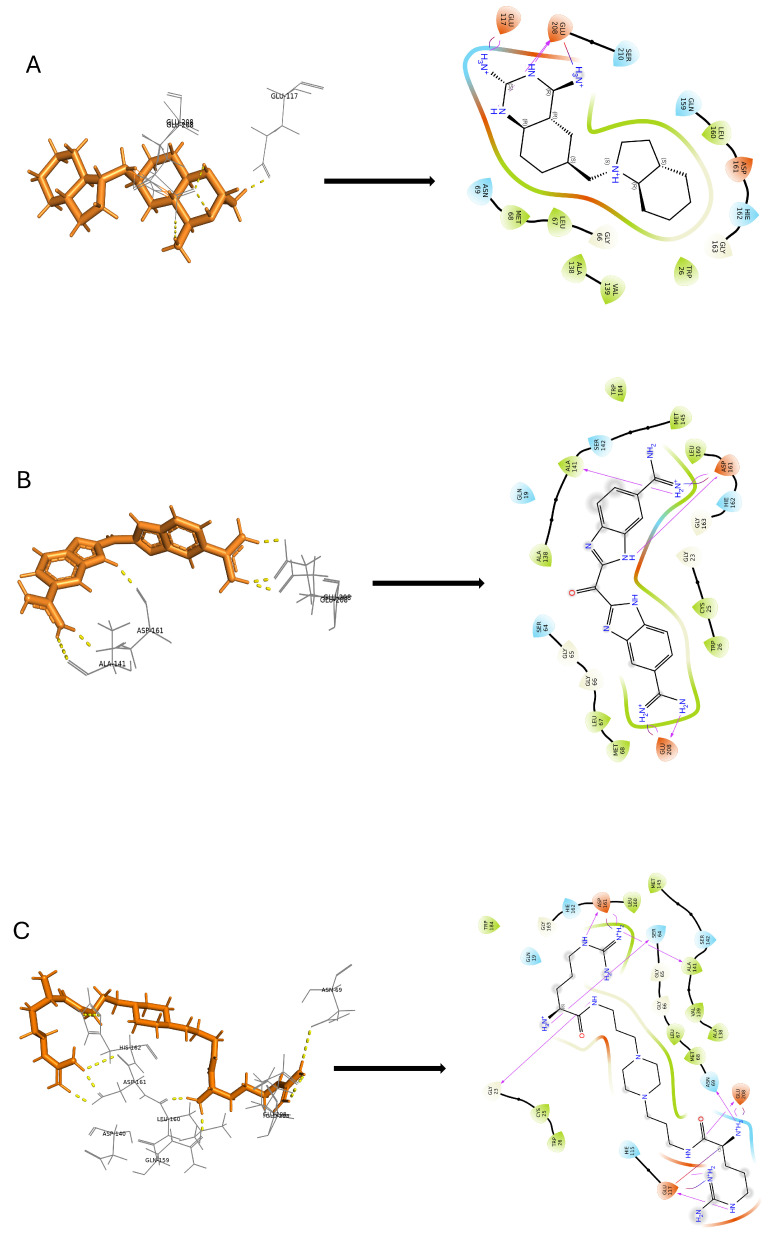
Deep learning modeling. Two-dimensional molecular interaction pattern of compounds (**A**) DB02559, (**B**) DB03213, and (**C**) DBQ5199 with cruzipain.

**Figure 7:**
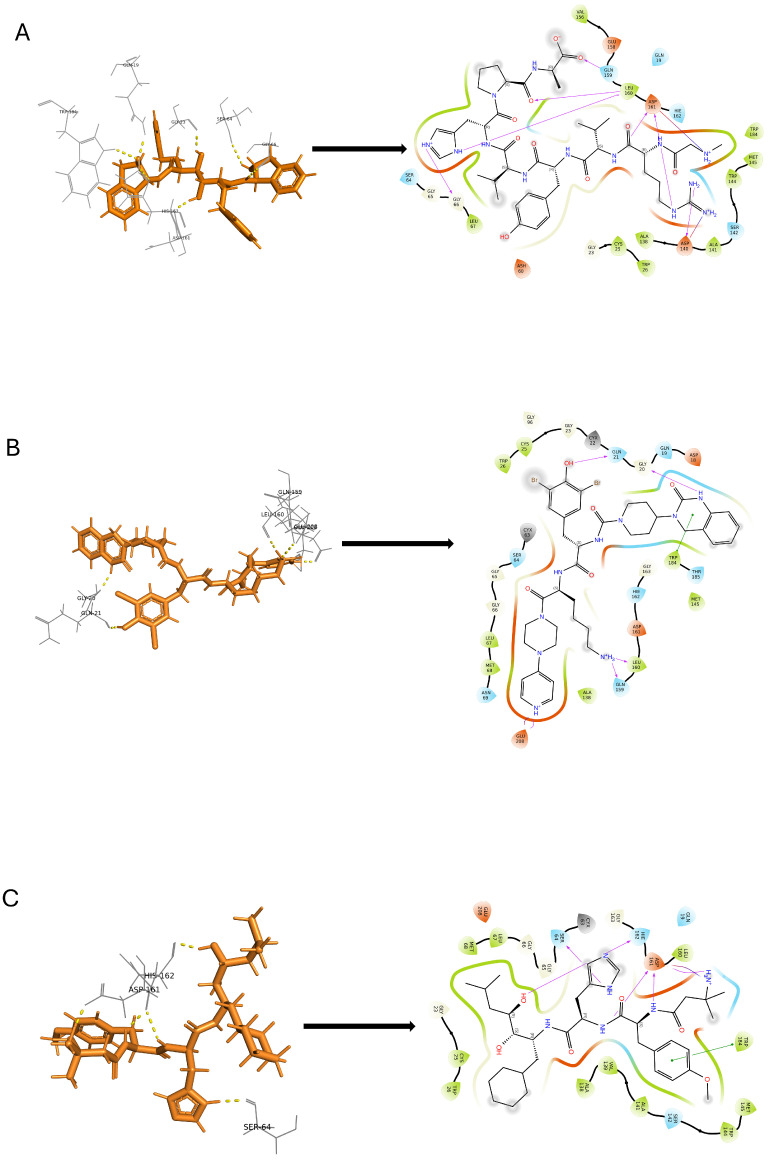
Pharmacophore modeling. Two-dimensional molecular interaction pattern of compounds (**A**) DB03395, (**B**) DB04869, and (**C**) DB02704, and with cruzipain.

The induced-fit docking (IFD) is a powerful approach that creates a variety of poses for the ligand complex, each of which includes distinct structural alterations to the receptor to match the ligand position, and then ranks these poses according to the Glide score to determine the optimal docked complex structure. In this investigation, we considered the top 10 docked compounds from both the pharmacophore and deep learning models for IFD docking. With the help of this method, the ideal ligand posture with the best score was found (Table S5). During the docking procedure, each ligand is assigned 10 possible poses and sorted by the docking score. The three top compounds from each outcome are selected as potential hits. However, the resulting hit compounds from deep learning had Glide scores of −9.253, −10.167, and −10.207 kcal/mol, as shown in Figure 6. Similarly, Figure 7 displays the top three substances from the pharmacophore model, which had Glide scores of −10.856, −11.177, and −13.286 kcal/mol, respectively. All hydrogen bond interactions identified for the top six compounds from both models are enlisted in Table 3. A potent technique known as induced-fit generates several ligand–receptor combinations while considering certain structural changes the receptor makes to receive the ligand. As a result, our comprehensive technique allowed us to identify the most promising ligand–receptor combinations for further study.

### 2.7. In Silico Predicted Physicochemical Parameters

Absorption, Distribution, Metabolism, and Excretion (ADME) are important factors in medicinal chemistry. To evaluate the drug-likeness of the top 10 hits from the docking study, we evaluated physio-chemical factors, such as Lipinski’s rule of five. The descriptors are depicted in Table 4. The molecules had a molecular weight higher than the limit. The Log Po/w values were predicted, and all values except for compounds DB15199 and DB06763 were within the recommended range of −2.0–6. However, except for DB15199, DB00183, DB04593, and DB06763, the Log BB (blood–brain partition coefficient) values for all substances are in the range of −3–1.2. Furthermore, these results suggest that the molecule may have suitable drug-like properties. The molecules show good solubility, as indicated by the Log S values, except for compounds DB15199, DB02559, DB02704, and DB04869. The human oral absorption of the compounds varied, with some having poor absorption and others having high absorption, as shown in the table. However, the compounds were found to have a high number of likely metabolic reactions. Overall, the results show fewer violations of the rules and are satisfactory.

### 2.8. MD Simulations

**Figure 8:**
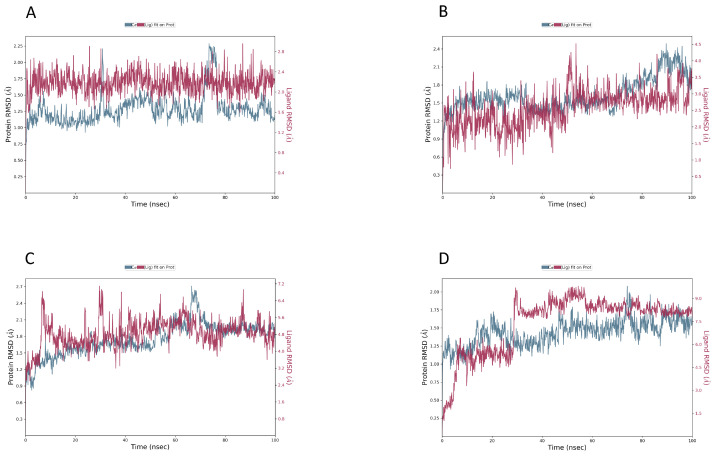
Shows the RMSD for compounds (**A**) DB02704, (**B**) DB03395, (**C**) DB03213, and (**D**) DB15199 with the protein cruzipain.

The stability and binding mechanisms of selected compounds (DB02704, DB03395, DB03213, DB15199) in complex with T.(cruzi) cruzipain were evaluated using molecular dynamics simulations. The MD simulation of cruzipain protein and the CHEMBL chemical system was qualitatively analyzed, with an emphasis on RMSD, RMSF, and hydrogen bond analyses. However, the stability of the protein–ligand complex was assessed using the RMSD and RMSF of the unbound protein structure. Throughout the simulation, lower RMSD values indicate that the protein–ligand combination is more stable. Similarly, Figure 7 demonstrates the compactness, fit, and stability of all compounds within the allosteric site. The values of the root mean square deviation (RMSD) derived from the trajectory analysis range from 1.0 to 3.0. The most stable compound was DB02704, which promptly reached a stable state with a 2.00 Å RMSD and maintained it throughout the experiment with just small variations. After 20 ns, DB03395 achieved stability with a little reduced but still acceptable level. After DB03213 stabilized, it exhibited small oscillations, which took around 50 nanoseconds to settle. During the experiment, DB15199 showed a slight RMSD divergence between 70 and 80 ns. Nevertheless, it stabilized towards the end, as displayed in Figure 8.

**Figure 9:**
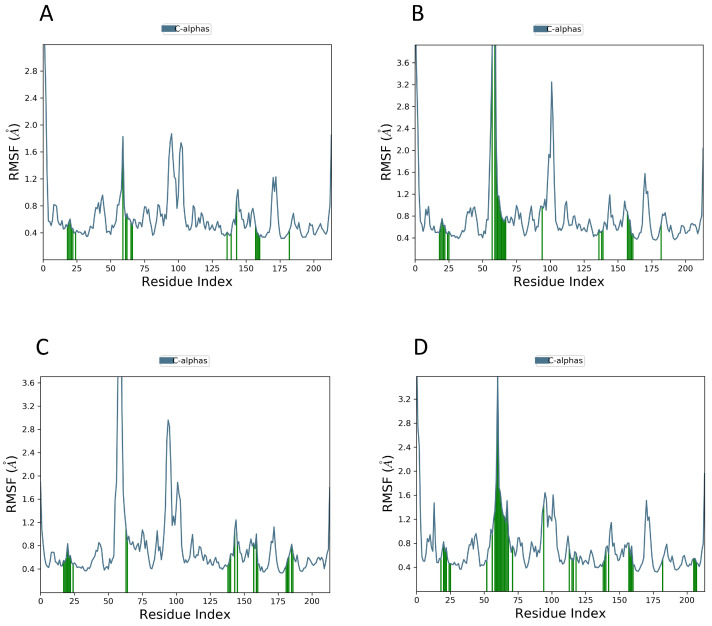
Shows the RMSF for compounds (**A**) DB02704, (**B**) DB03395, (**C**) DB03213, and (**D**) DB15199 with protein cruzipain.

In the RMSF graph, some residue segments showed slight differences, but the majority stayed below 0.7 nm, indicating that a stable simulation is shown in Figure 9. It is worth noting that changes up to 3.6 were mainly observed in residues with a concentration between 50 ns and 100 ns. This area contains crucial active site residues and a few loop sections of the protein.

**Figure 10:**
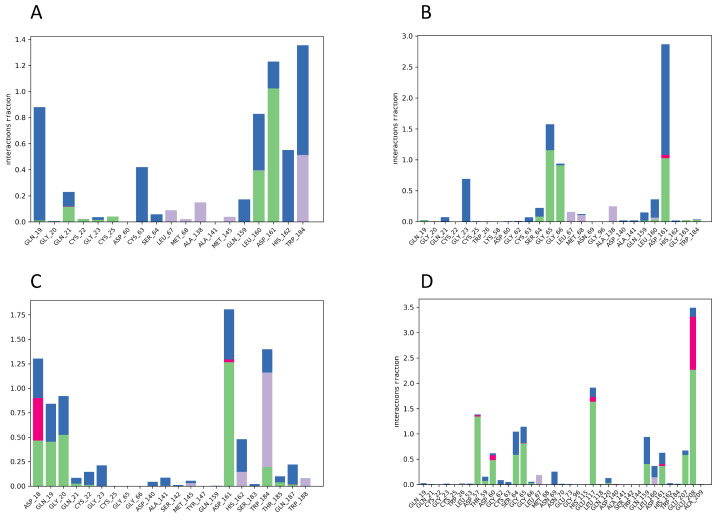
Shows the count of the interaction fraction in histogram form for compounds (**A**) DB02704, (**B**) DB03395, (**C**) DB03213, and (**D**) DB15199 with protein cruzipain.

**Figure 11:**
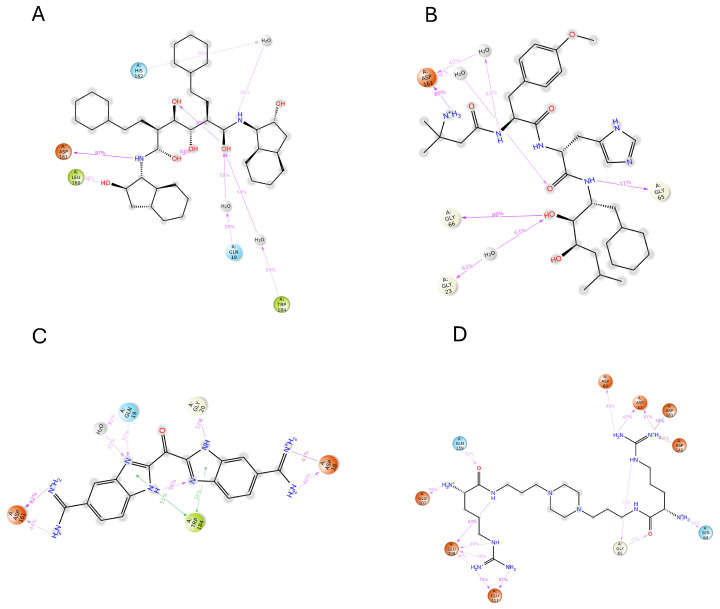
Two-dimensional structures show the count of the interaction fraction for compounds (**A**) DB02704, (**B**) DB03395, (**C**) DB03213, and (**D**) DB15199 with protein cruzipain.

In DB15199, amino acid ASP 161 has the highest interaction percentage of 1.25 and is highlighted in green, showing the most hydrogen bonds among all the complexes represented in Figure 10 and Figure 11. These hydrogen bonds are crucial for effective ligand binding. Moreover, it can be observed that ASP 161 is accompanied by various residues (GLY 65-66, LEU 160, LEU 53, GLU 117, and GLU 208) that display different types of hydrogen bonding. It is noteworthy that ASP 161 keeps up consistent contact throughout the investigation.

Subsequently, the protein residues interacting with the ligand are highlighted by the green vertical bars. With the largest changes up to 2.4 Å, the area between residues (100–130) shows the most notable alterations among these interactions. In addition, TRP184-188, ALA 138, PHE 404, and LEU 67 form hydrophobic interactions in the active pocket, where the molecule is positioned carefully. Thus, we concluded that, during the 100 ns MD simulation, no significant changes were observed at the TCP ligand binding site. The TCP–ligand complexes remained stable throughout the simulation, without any dramatic alterations. Furthermore, there was no unexpected activity observed in the complexes, which indicates that the virtual hit substances were successfully bound to the active region of the TCP protein.

## 3. Discussion

In this study, we attempted to address the problem of developing better treatments for Chagas disease, an illness that affects millions of individuals globally. While there are drugs like nifurtimox and benznidazole that are accessible, they have disadvantages such as side effects and a decreasing level of effectiveness with time. CZP, a crucial enzyme in the parasite that causes Chagas disease, was the focus of our investigation. We used modern tools, such as deep learning and computer simulations, to sort through the vast Drug Bank database. Our objective was to find drugs that could inhibit CZP. Following extensive testing and analysis, we were able to identify four compounds (DB02704, DB03395, DB03213, and DB15199) that exhibited significant potential in their interactions with CZP, indicating that they might be useful therapeutic choices. This work reveals how current medications might be repurposed to treat Chagas disease and shows the influence of computational approaches in drug discovery with a high success rate. In the future work, we will perform lengthier molecular dynamics simulations for the resulting four hit compounds. Additionally, the final compound will undergo an energy calculation using teh advanced Molecular Mechanics/Poisson–Boltzmann Surface Area (MMPBSA) analytical tool. Moreover, experimental validation will be conducted on the hit compounds to investigate their inhibitory effects against the cruzipain protein. This comprehensive process is necessary to evaluate the safety profile and efficacy of the identified pharmaceutical candidates before considering any potential clinical applications.

## 4. Materials and Methods

This section contains information on the dataset retrieval and preprocessing for both models such as QSAR and deep learning. On the other hand, the QSAR, Pharmacophore, and deep learning approaches have been extensively described.

### 4.1. Data Retrieval for Deep Learning

The benchmark dataset utilized in this research was sourced from the CHEMBL database [21] (refer to this link: https://www.ebi.ac.uk/chembl as accessed on 2 July 2023), which included 204 inhibitor compounds (supplementary file). These inhibitors have IC_50_ values less than or equal to 1000 nM, indicating that they are active compounds, as determined by a variety of published bioassays [6,22,23,24,25,26,27,28,29,30,31,32,33,34,35,36,37,38,39,40,41,42,43,44,45,46,47,48]. The CHEMBL IDs for the compounds, canonical smiles, and corresponding IC_50_ values were carefully organized into a comprehensive dataset, resulting in a data frame table that encapsulates each constituent along with its associated bioactive classification. To facilitate subsequent analyses, the IC_50_ measurements have been converted to their negative logarithmic equivalents through the following formula:

(1) $$pIC_{50}=-\log\left({\mathrm{IC}}_{50}\times 10^{-9}\right)$$

Simultaneously, the amino acid sequence of the target protein *T. cruzi* cruzipain (PDB: 3IUT), was downloaded in FASTA format from the Protein Data Bank (PDB) [49]. During the final phases of data compilation, the amino acid sequences of cruzipain were aligned with each active molecule, by their respective index positions and pIC_50_ values. The random dataset splitting technique has been adopted to split the dataset in the three subsets, namely the training, validation, and testing subsets, each constituting 70%, 10%, and 20%, respectively. The deep learning model was trained on 70% of the dataset and validated on 10% of the dataset. Observing the standard deviation between the training and validation results during the training process to prevent overfitting. Apart from that, to ensure the generalizability of the deep learning model, the model was tested on 20% of unseeded test dataset.

### 4.2. Data Retrieval for QSAR

The small molecules used in this study were taken from the literature with their IC_50_ value [27] shown in Supplementary Table S1. A total of 36 compounds were retrieved from PubChem (https://pubchem.ncbi.nlm.nih.gov/ as accessed on 15 July 2023) database. Compounds’ 2D structures were optimized using the LigPrep module offered by Schrodinger 2023-2 [50]. However, the optimization method used default settings, with the OPLS4e force field applied and ionization states neutralized. The intrinsic stereoisomerism of each ligand including its critical chirality was preserved throughout this optimization process. Following optimization, the structures were aligned using the ligand alignment tool. Since every structure is a congeneric series, the software algorithm was used to automatically determine the reference scaffold for the alignment. This is helpful because the program identifies the best scaffold, which serves as a guide. Meanwhile, the biological activity values (IC_50_) were converted into pIC_50_ using the above-mentioned Equation (1). Finally, the prepared structures were employed for 3D-QSAR and pharmacophore modeling.

### 4.3. Building 3D-QSAR Model

In this 3D-QSAR study, we built a model using an optimized and aligned set of compounds. The dataset was randomly divided with a ratio of 70:30 resulting in 27 compounds as training and 9 compounds as the testing set. After a thorough inspection of the training and test set data, both sets had low, medium, and high active data, which was necessary to build an appropriate model. The phase module embedded in the Schrodinger suit was used to build a 3D field-based QSAR model. Our technique includes the use of Gaussian or COMSIA-like models built on a grid with many points arranged in a rectangle pattern. However, all the fields were identified by concatenating and adding atom-level chemical descriptions. The fields were estimated using a Gaussian function that considers the distance between atoms and places on the rectangular grid. Similarly, an atomic radius was used to compute steric values, while partial atomic charges and predicted values of AlogP (octanol-water partition coefficient) were used to generate electrostatic fields. A partial least squares (PLS) analysis was performed using a total of five factors to assist robust modeling. To facilitate the prediction for compounds larger than those in the training set, the grid spacing was set at 1.0 Å and increased by 3.0 Å beyond the limit of the training set. Furthermore, force field contributions from atoms within a radius of 2.0 Å were eliminated from the training dataset. This will help prevent fields closer to the nucleus from dominating. However, the cut-off threshold value was set to 30 kcal/mol to truncate steric and electrostatic fields. Variables with standard deviations smaller than 0.01 were removed to improve the models’ stability. In particular, the goal of our work was to methodically create a reliable 3D-QSAR model that could predict the compound behavior outside of the training dataset.

### 4.4. Ligand-Based Pharmacophore Generation

The phase [50] module of the Schrodinger suite is a crucial tool for understanding pharmacophores. This widely used method makes it possible to fully analyze the chemical characteristics of active sites as well as the three-dimensional spatial configurations of ligand substituents. Notably, the phase module has six in-built pharmacophore features: hydrogen bond donor (D), hydrogen bond acceptor (A), hydrophobic group (H), aromatic ring (R), negative ionizable group (N), and positive ionizable group (P). Using a tree-based partition technique, the common pharmacophore features shared by all the compounds’ investigative conformers were utilized to build pharmacophore sites. Based on the pIC_50_ values, the ligands were classified as active or inactive in the context of an experimental data analysis. Those compounds, having a pIC_50_ value equal to or more than 6.1, were categorized as active, while those with a pIC_50_ value less than 6.1 were classified as inactive. The hypothesis was set to match at least 85% of the active compounds in the inner setting. However, the number of characteristics allowed in the hypothesis was limited to a minimum of 5 and a maximum of 6. A criterion of 0.5 was established for the recognition of the differences in the hypotheses. Throughout the analytical process, an excluded volume shell was created to account for regional differences. In conclusion, the study aimed to identify active and inactive ligands by analyzing their pIC_50_ values.

### 4.5. Pharmacophore Validation

Pharmacophore validation was performed to determine whether our models were accurate enough to predict the active chemicals. For this purpose, we built a Deep decoy dataset (https://github.com/oxpig/DeepCoy as accessed on 6 August 2023), generating property-matched decoy molecules using a deep learning tool known as deep coy [51], to validate the highest-scoring hypothesis, used for virtual screening against the merged decoy-active dataset obtained in the previous step. In this process, we took the active molecules’ SMILES and generated 40 inactive decoy structures for every single active molecule. Following that, an enrichment factor was computed for the selected hypothesis. In addition, the default rejection criteria were defined.

### 4.6. Deep Learning Model

According to the current trend, we employed the deep learning model known as DeepPurpose for the virtual screening of the drug bank database. DeepPurpose, an advanced deep learning library designed for predicting Drug–Target Interaction (DTI) [20] is within our study. The code for DeepPurpose is publicly available at https://github.com/kexinhuang12345/DeepPurpose as accessed on 8 August 2023. The Simplified Molecular Input Line Entry System (SMILES) representations of molecules and the amino acid sequences of genomes that code for proteins are the inputs used by this library. DeepPurpose utilized various encoders to generate embeddings for both compounds and proteins. These embeddings are then concatenated and fed into a multi-layer perceptron, which predicts the binding affinity. DeepPurpose offers a selection of pre-trained models that are readily accessible for analysis. It has fourteen models in all, differentiated by the DTI training dataset and the encoders used in each. In our research, we avoided pre-trained models and trained our model using a newly constructed dataset for a specific disease. For drug encoding, we used a range of techniques, including convolutional neural networks (CNNs), daylight fingerprints, Morgan fingerprints, and message-passing neural networks (MPNNs). Protein encoding comprised amino acid composition (AAC) and CNN-based methods. We created an environment for DeepPurpose implementations using Python 3.6 and Jupyter Notebook v7.0.4. The core deep learning network implementation utilized PyTorch 1.4. The RDKit library (https://www.rdkit.org/docs/index.html as accessed on 26 September 2023) was used to generate fingerprints and Morgan fingerprints from compounds.

### 4.7. Hyper Parameterization

The configuration of hyperparameters plays an essential role in improving the performance of deep learning models. In the context of hyperparameter configuration, the dimensions allocated to the three hidden layers were specified as follows: 1024, 1024, and 512. The training phase lasted 400 epochs, and each iterative training cycle included both forward and backward passes. Notably, the learning rate was fixed at 0.001, a highly significant parameter considering its significant impact on the length of the learning process and the optimization result. It is imperative to exercise prudence in the selection of this value, as an excessively large or exceedingly small learning rate can yield undesirable results. The number of batches processed per epoch, often referred to as the batch size, was configured at 14 for each training epoch. Additionally, a 128-dimensional vector space was employed to represent the chemical structure data. In the realm of protein target sequence filtering, three convolutional layers were implemented with dimensions of 32, 64, and 96. For the convolutional neural network (CNN) utilized in the targeting process, kernels with dimensions of 4, 8, and 12 were employed. The deep learning model training was conducted using an NVIDIA TITAN Xp GPUs with 12GB and RAM is 250GB. Parallel processing was implemented across three GPUs using CUDA version 11.6. The detailed information of the GPUs was enlisted in the Supplementary Figure S1. The optimized hyperparameters for compounds and proteins were enlisted in Supplementary Table S2.

### 4.8. Virtual Screening

#### 4.8.1. Drugbank Database Preparation

Explaining a target database carefully is the first and most crucial step in performing virtual screening. However, in the current inquiry, considerable attention is paid to using the Drugbank database for screening purposes. These database compounds were downloaded in SDF format from the official Drug Bank repository (https://go.drugbank.com/ as accessed on 2 July 2023), resulting in a total of 11,565 unique pharmaceutical drugs. The dataset underwent a refinement process to remove compounds containing metal ions with additional attention to those lacking a valid SMILES notation. After careful processing, we assembled a collection of 8533 molecules for further research.

#### 4.8.2. Deep Learning-Based Virtual Screening

The deep learning model was thoroughly validated before being deployed for virtual screening. After training the model, a dataset of 8533 chemicals was passed for virtual screening. Based on the estimated bioactivity value of each molecule, we set up the framework to only return the top-scored compounds.

#### 4.8.3. Pharmacophore-Based Virtual Screening

Following the satisfactory validation of the top pharmacophore model, it was virtually screened against the dataset of 8533 chemicals from the drug bank. However, before screening, all the dataset molecules were properly minimized and converted to 3D structures using the MacroModel tool of Schrodinger 2023-2 [52]. Furthermore, the prepared molecules were subjected to the screening process. For screening purposes, based on the phase-hypo score, the most notable pharmacophore similarities matched were selected.

### 4.9. Docking Investigation

#### 4.9.1. Glide SP (Standard Precision) Docking

In the process of conducting docking studies, the compounds obtained during the previous screening step were utilized. In brief, a Cruzipain (CZP) 3D crystal structure (PDB: 3IUT) was obtained from the online repository Protein Data Bank (PDB) (https://www.rcsb.org/ as accessed on 15 July 2023). Before docking, the structure of the protein was prepared using a protein preparation workflow embedded in Schrodinger’s maestro tool [53]. In Protein preprocessing, all the water molecules and the co-crystal ligands were removed and minimized to remove the steric clashes using the OPLS4 force field. The ligand binding coordinates were taken from the literature [52]. A receptor Grid was generated around the protein (X = 4.24, Y = 8.8, Z = 10.12, 30 × 30 × 30) by choosing the active site residues. Additionally, The van der Waals radius of the receptor atoms was scaled to 1.00 with a partial atomic charge of 0.25, and the centroid of the ligand was chosen to construct a grid box around it. Similarly, the drug-like molecules were prepared, and energy was minimized by using the LigPrep module. Subsequently, parallel docking analysis was performed on the resulting compounds from the virtual screening of both deep learning and pharmacophore models. The GLIDE was set to reject ligands having more than 500 atoms and rotatable bonds [54]. To provide an acceptable level of precision, the subsequent docking process was carried out in standard-precision mode. Moreover, nitrogen inversion and ring conformation were considered while including the flexibility in the ligand sampling procedure. Notably, a sample bias was applied to all torsions associated with assigned functional groups, which added a strategic compound to the study. State fines were incorporated into the computations using the Epik tool [55] to optimize the final docking score. Following the docking process, the obtained poses were carefully examined to see which ones had favorable root-mean-square deviation (RMSD) refinement values that aligned with the native ligand binding mode. Then, the most promising poses that showed a strong resemblance to the native ligand binding mechanism were carefully chosen for additional study. A post-docking minimization phase was also performed, which only involved evaluating ten poses for each ligand molecule to determine the most beneficial conformation.

#### 4.9.2. Induced Fit Docking

The induced fit docking module of Schrodinger was utilized to accomplish induced fit docking [56,57]. For redocking, we used the best score, docked compounds from both models, pharmacophore, and deep learning. Utilizing the previously constructed receptor grid box, these compounds go through induced fit docking. Regarding the glide and induced-fit score values of the individual chemicals, there was hardly any obvious variation. However, it is significant that we decided without performing the first docking setup since the ranking had already been performed. The sample ring conformation with an energy window of 2.5 kcal/mol was picked for the conformational sampling option. Furthermore, the receptor van der Waals scaling and the ligand van der Waals scaling were set to 0.7 and 0.5, respectively. The residues were refined within 5 Å of the ligand poses, and Glide redocking was performed on the structures within 30 kcal/mol of the best structure. Supplementary Table S5 provides a thorough comparison of all the produced parameters for the hit compounds that were found. According to the study’s conclusions, the Glide method’s effectiveness was highlighted in precisely filtering and finding the best chemicals, thereby reducing false negatives for both hit compounds.

### 4.10. Absorption, Distribution, Metabolism, Excretion (ADME), and Toxicity

One of the key considerations in turning a molecule into a medication is evaluating a molecule’s characteristics of absorption, distribution, metabolism, and excretion (ADME) [58]. Computer-based prediction is crucial for early drug candidate selection due to clinical trial demands. For this purpose, the QikProp module in Schrodinger is utilized to evaluate the ADME properties such as BBB permeability, surface area, percentage of oral absorption, etc., of the selected compounds [59,60]. The resulting compounds following redocking go through ADMET screening before being employed in molecular dynamic modeling. Additionally, these drug-like compounds were sent in SDF format to QikProp for screening. However, all the compounds followed the five rules of Lipinski, and the results were produced in a CSV file.

### 4.11. Molecular Dynamics Simulation

Molecular dynamics (MD) study was performed using the Desmond module of the Schrodinger software (Maestro version 13.6.122, Release 2023-2) for the best compounds obtained from the induced-fit docking [61]. In an orthorhombic box of size 10 Å × 10 Å × 10 Å, the ligand–protein combination was built up for the simulation utilizing a water model box (TIP3P) as the solvent. Additionally, counter-ions were added, followed by a minimization step, to keep the system neutral. Also, the salt content was adjusted to 0.15 M Na+ with Cl- ions to approximate the physiological circumstances. At a temperature of 300 K and a pressure of 1.63 bar, 100 ns of molecular dynamics simulations were run in the NPT ensemble [62]. While trajectory data were taken every 100.0 picoseconds, energy data were captured at intervals of 10 picoseconds. The OPLS4 force field was used to perform these simulations. After the completion of the simulation, the Maestro Desmond simulation interaction diagram tool was used to create plots and figures showing the results.

## 5. Conclusions

In this study, we used both conventional and innovative computer-aided drug design approaches to identify the novel inhibitors of cruzipain. The QSAR models were carefully verified for predicted accuracy and showed good stability. In addition, we used a deep learning model that mimics human neural processes. This deep learning system, which includes internal molecular encoders, eliminates the need for external descriptor calculators, making it distinct from typical QSAR models. In addition, in line with our deep learning-based method, we developed a pharmacophore model. These two virtual screening methods aided in the screening of a Drugbank database, offering useful insights for future molecular docking analysis. Our thorough analysis revealed many functional groups that are essential for interactions within the target protein’s binding region. We believe that the most promising chemical identified using this technology may exhibit inhibitory properties, hence requiring additional in vitro research to fully understand its potential therapeutic effects.

## Figures and Tables

**Table 1:** Statistical evaluation of the QSAR model.

Factors	SD	$r^{2}$	$r^{2}$ Scramble	F	P	RMSE	Pearson-R
1	0.4866	0.4710	0.2810	24.0	3.96 $\times \;10^{-5}$	0.46	0.7224
2	0.4514	0.5614	0.4323	16.6	2.22 $\times \;10^{-5}$	0.41	0.8994
3	0.4245	0.6272	0.5292	14.0	1.46 $\times \;10^{-5}$	0.42	0.8665
4	0.3900	0.6978	0.6215	13.9	5.44 $\times \;10^{-5}$	0.41	0.8790
5	0.3753	0.7318	0.6854	12.6	5.98 $\times \;10^{-6}$	0.40	0.8564

**Table 2:** Pharmacophore models and their score.

Hypo ID	Phase Hypo Score	Vector Score	Volume Score	BEDROC Score	Survival Score
AAAHR_1	1.279	1.000	0.872	0.950	5.490
AAAHR_3	1.259	0.976	0.872	0.938	5.347
AAAHR_2	1.256	0.976	0.872	0.935	5.357
AAADHR_3	1.226	0.983	0.872	0.886	5.672
AADHR_2	1.223	0.998	0.872	0.895	5.467

**Table 3:** Docking score analysis and the molecular interaction of compounds from induced-fit docking.

S. No	Drugbank ID	Compound Name	H-Bond Interaction	IFD Score (Kcal/mol)
1	DB03213	Bis(5-Amidino-2-Benzimidazolyl) Methane Ketone	ASP-161, GLU-208	−10.167
2	DB02559	6-(Octahydro-1h-Indol-1-Ylmethyl) Decahydroquinazoline-2,4-Diamine	GLU-208	−10.207
3	DB15199	Ciraparantag	ASP-161, GLY-23, SER-64, GLU-117, GLU-208, ASN-69	−9.253
4	DB02704	(2R,3R,4R,5R)-3,4-Dihydroxy-N,N’-bis [(1S,2R)-2-hydroxy-2,3-dihydro-1H- inden-1-yl]-2,5-bis(2-phenylethyl) hexanediamide	ASP-161, SER-64, GLY-66, GLY-23, GLY-19	−11.177
5	DB03395	Enalkiren	ASP-161, HIE-162, SER-64	−10.856
6	DB04869	Olcegepant	GLN-159, GLN-21, GLY-20	−13.285

**Table 4:** The absorption, distribution, metabolism, excretion, and toxicity (ADMET) of the final compounds from docking.

Drugbank ID	Stars	Mol. MW	Dipole	SASA	Donor HB	Accpt HB	QPlog o/w	QPlogS	QPlog Khsa	No. of Metabolites	QP Log BB	%Human Oral Absorption
DB03213	4	346.351	5.974	613.698	8	8	−0.602	−2.828	−0.731	0	−3.358	24.921
DB15199	13	512.7	3.277	1008.917	14	15	−4.661	2	−1.966	12	−6.172	0
DB01705	1	332.367	8.009	612.184	8	6	0.24	−2.932	−0.606	1	−2.903	37.431
DB02559	2	307.481	1.904	589.065	6	7	−1.371	2	−0.169	3	−0.237	19.966
DB00183	11	767.896	9.505	1157.206	4.75	13.25	3.256	−5.197	−0.768	9	−4.437	2.862
DB02704	10	648.797	3.572	1056.131	4	9.8	5.862	−7.727	0.569	12	−2.4	63.601
DB03395	7	656.864	1.331	1066.353	5.5	11.65	3.509	−4.657	−0.137	9	−2.333	34.644
DB04593	8	587.693	6.042	984.581	8.5	13	0.868	−4.599	−1.142	6	−5.243	0
DB04869	9	869.655	6.464	1147.692	4.5	12.25	4.49	−7.99	0.52	10	−2.443	29.794
DB06763	17	912.057	14.239	1258.121	9.5	21.25	−3.498	−2.531	−2.55	14	−5.912	0

## Data Availability

The datasets and code used during the current study are publicly available for download at (https://github.com/waleed551/Cruzipain as accessed on 1 February 2024). The data are accessible to the public without any restrictions, promoting transparency and facilitating the reproducibility of the study findings. Supplementary file S1: The compounds used for the QSAR model are listed in the Table S1. Table S2 includes the hyperparamaters for the deep learning. Tables S3–S5 contain the docking results. Supplementary file S2: The dataset used for deep learning training is available in csv format.

## Supplementary Materials

The following supporting information can be downloaded at: https://www.mdpi.com/article/10.3390/ijms25073747/s1.

## Abbreviations

The following abbreviations are used in this manuscript:

*T. cruzi*	Trypansoma cruzi
CZP	Cruzipain
QSAR	Quantitative structure–activity relationship
CNN	Convolutional neural networks
MPNN	Message-passing neural networks
AAC	Amino acid composition
ADME	Absorption, distribution, metabolism, and excretion
SMILES	Simplified Molecular Input Line Entry System
DTI	Drug target interaction
AlogP	Octanol–water partition coefficient
FASTA	Fast alignment
