# Supplementary material for: Integrated Computational Approaches for Drug Design Targeting Cruzipain
Source: Int J Mol Sci. 2024 Mar 27;25(7):3747. doi: 10.3390/ijms25073747 (PMC11011879; doi:10.3390/ijms25073747)
Supplement: Supplementary file 1 [file ijms-25-03747-s001.zip › Supplementary file S1.pdf]

Table S1. Compounds for QSAR taken from literature.

No	Compounds	SMILES
1	CRUZAIN-IN-1	CCn1cnc2c(Nc3cc(F)cc(F)c3)nc(C#N)nc21
2	6-(3-Chloroanilino)-9-cyclopentyl-2-purinecarbonitrile	N#Cc1nc(Nc2cccc(Cl)c2)c2ncn(C3CCCC3)c2n1
3	6-(3-Chloroanilino)-9-ethyl-2-purinecarbonitrile	CCn1cnc2c(Nc3cccc(Cl)c3)nc(C#N)nc21
4	4-(3,5-Difluoroanilino)-6-(2,2-difluoroethylamino)-1,3,5-triazine-2-carbonitrile	N#Cc1nc(NCC(F)F)nc(Nc2cc(F)cc(F)c2)n1
5	4-(Cyclopentylamino)-6-(3-nitrophenylamino)-1,3,5-triazine-2-carbonitrile	N#Cc1nc(Nc2cccc([N+](=O)[O-])c2)nc(NC2CCCC2)n1
6	4-(3-Chlorophenylamino)-6-(cyclopentylamino)-1,3,5-triazine-2-carbonitrile	N#Cc1nc(Nc2cccc(Cl)c2)nc(NC2CCCC2)n1
7	4-(3-Bromophenylamino)-6-(cyclopentylamino)-1,3,5-triazine-2-carbonitrile	N#Cc1nc(Nc2cccc(Br)c2)nc(NC2CCCC2)n1
8	4-(Cyclopentylamino)-6-(3,5-difluorophenylamino)-1,3,5-triazine-2-carbonitrile	N#Cc1nc(Nc2cc(F)cc(F)c2)nc(NC2CCCC2)n1
9	4-(Cyclopentylamino)-6-(3-fluorophenylamino)-1,3,5-triazine-2-carbonitrile	N#Cc1nc(Nc2cccc(F)c2)nc(NC2CCCC2)n1
10	4-(Cyclopentylamino)-6-(3,5-dichlorophenylamino)-1,3,5-triazine-2-carbonitrile	N#Cc1nc(Nc2cc(Cl)cc(Cl)c2)nc(NC2CCCC2)n1
11	Cruzain-IN-1	CCn1cnc2c(Nc3cc(F)cc(F)c3)nc(C#N)nc21
12	6-(3,5-Difluoroanilino)-9-(2,2-difluoroethyl)purine-2-carbonitrile	N#Cc1nc(Nc2cc(F)cc(F)c2)c2ncn(CC(F)F)c2n1
13	9-cyclopentyl-6-(3,5-difluorophenylamino)-9H-purine-2-carbonitrile	N#Cc1nc(Nc2cc(F)cc(F)c2)c2ncn(C3CCCC3)c2n1
14	6-(3-Chloroanilino)-9-ethyl-2-purinecarbonitrile	CCn1cnc2c(Nc3cccc(Cl)c3)nc(C#N)nc21
15	4-(3,5-Difluoroanilino)-6-(2,2-difluoroethylamino)-1,3,5-triazine-2-carbonitrile	N#Cc1nc(NCC(F)F)nc(Nc2cc(F)cc(F)c2)n1
16	4-(Cyclopentylamino)-6-(m-tolylamino)-1,3,5-triazine-2-carbonitrile	Cc1cccc(Nc2nc(C#N)nc(NC3CCCC3)n2)c1
17	4-(Cyclopentylamino)-6-(4-fluorophenylamino)-1,3,5-triazine-2-carbonitrile	N#Cc1nc(Nc2ccc(F)cc2)nc(NC2CCCC2)n1

18	4-(Cyclopentylamino)-6-(3-(trifluoromethyl) phenylamino)-1,3,5-triazine-2-carbonitrile	N#Cc1nc(Nc2cccc(C(F)(F)F)c2)nc(NC2CCCC2)n1
19	6-(3-Chloroanilino)-9-cyclopentyl-2-purinecarbonitrile	N#Cc1nc(Nc2cccc(Cl)c2)c2ncn(C3CCCC3)c2n1
20	6-(2,2-difluoroethylamino)-9-(3,5-difluorophenyl)-9H-purine-2-carbonitrile	N#Cc1nc(NCC(F)F)c2ncn(-c3cc(F)cc(F)c3)c2n1
21	4-(Biphenyl-3-ylamino)-6-(cyclopentylamino)-1,3,5-triazine-2-carbonitrile	N#Cc1nc(Nc2cccc(-c3ccccc3)c2)nc(NC2CCCC2)n1
22	4-(4-Bromophenylamino)-6-(cyclopentylamino)-1,3,5-triazine-2-carbonitrile	N#Cc1nc(Nc2ccc(Br)cc2)nc(NC2CCCC2)n1
23	9-(3-chlorophenyl)-6-(ethylamino)-9H-purine-2-carbonitrile	CCNc1nc(C#N)nc2c1ncn2-c1cccc(Cl)c1
24	4-(Cyclopentylamino)-6-(3,5-difluorophenylamino)-1,3,5-triazine-2-carbonitrile	N#Cc1nc(Nc2cc(F)cc(F)c2)nc(NC2CCCC2)n1
25	4-(Cyclopentylamino)-6-(3-nitrophenylamino)-1,3,5-triazine-2-carbonitrile	N#Cc1nc(Nc2cccc([N+](=O)[O-])c2)nc(NC2CCCC2)n1
26	4-Amino-6-(diphenylamino)-1,3,5-triazine-2-carbonitrile	N#Cc1nc(N)nc(N(c2ccccc2)c2ccccc2)n1
27	9-(3-chlorophenyl)-6-(2,2-difluoroethylamino)-9H-purine-2-carbonitrile	N#Cc1nc(NCC(F)F)c2ncn(-c3cccc(Cl)c3)c2n1
28	4-(3-Chlorophenylamino)-6-(cyclopentylamino)-1,3,5-triazine-2-carbonitrile	N#Cc1nc(Nc2cccc(Cl)c2)nc(NC2CCCC2)n1
29	4-(Cyclopentylamino)-6-(3-fluorophenylamino)-1,3,5-triazine-2-carbonitrile	N#Cc1nc(Nc2cccc(F)c2)nc(NC2CCCC2)n1
30	4-Ethoxy-6-piperidin-1-yl-1,3,5-triazine-2-carbonitrile	CCOc1nc(C#N)nc(N2CCCCC2)n1
31	4-(Cyclopentylamino)-6-(4-fluorophenylamino)-1,3,5-triazine-2-carbonitrile	N#Cc1nc(Nc2ccc(F)cc2)nc(NC2CCCC2)n1
32	4-(Cyclopentylamino)-6-(m-tolylamino)-1,3,5-triazine-2-carbonitrile	Cc1cccc(Nc2nc(C#N)nc(NC3CCCC3)n2)c1
33	9-(3,5-Difluorophenyl)-6-(ethylamino)-2-purinecarbonitrile	CCNc1nc(C#N)nc2c1ncn2-c1cc(F)cc(F)c1
34	6-(cyclopentylamino)-9-(3,5-difluorophenyl)-9H-purine-2-carbonitrile	N#Cc1nc(NC2CCCC2)c2ncn(-c3cc(F)cc(F)c3)c2n1

35	4-(Cyclopentylamino)-6-(3,5-dichlorophenylamino)-1,3,5-triazine-2-carbonitrile	N#Cc1nc(Nc2cc(Cl)cc(Cl)c2)nc(NC2CCCC2)n1
36	4-(Ethylamino)-6-(phenylamino)-1,3,5-triazine-2-carbonitrile	CCNc1nc(C#N)nc(Nc2ccccc2)n1

Table S2. Deep learning model Hyper-parameters optimal values and range.

Hyper-parameters	Range	optimal
CNN filters	16 -512	[32,64,96]
CNN kernels	4-16	[4,8,12]
Learning rate	0.1 -0.0005	0.001
hidden_dim_drug	32 - 256	128
mpnn_hidden_size	32-126	128
mpnn_depth	2-9	3

Table S3. Docking score analysis and molecular interaction of compounds from Deep learning

S.No	H-bond interaction	Glide score (Kcal/mol)
DB01705	ASP-161, LEU-160,	-8.057
DB03874	ASP-18	-6.977
DB15199	GLU-117	-9.394
DB02559	ASP-161, TRP-184	-7.516
DB03213	GLY-20, ASP-18,	-7.409
DB04710	GLY-66, ASP-161	-7.087

DB09012	GLY-66, ASP-161	-6.857
DB04771	ASP-18, ASP-161	-7.524
DB04613	GLN-19, ASP-161	-7.375
DB06841	ASP-161, GLY-66	-7.579

Table S4. Docking score analysis and molecular interaction of compounds from Pharmacophore modeling.

S.No	H-bond interaction	Glide score (Kcal/mol)
DB00183	GLY-66, GLN-19, ALA-141	-8.104
DB01167	GLY-66	-8.834
DB01846	ASN-69, GLU-208, GLN-19, ASP-18, TRP-184, GLY-66	-8.443
DB02704	LEU-160, ASP-161, GLN-19, GLN-21	-8.217
DB03395	ASP-161, SER-64, GLY-66	-8.141
DB03415	GLY-66, TRP-184, ASP-18, GLN-19, GLN-21	-8.223
DB04191	ASP-161, GLY-66, TRP-184, GLY-20, ASP-18	-8.563
DB04593	ASP-18, GLN-19, TRP-184, ASP-161, HIE-162	-9.126
DB04869	GLY-96	-10.362
DB06763	GLN-19, SER-64, GLU-117, GLN-120	-10.238

Table S5. Induced Fit Docking (redocking) for the top hit compounds.

S.no	Hydrophobic interaction	H-bond interaction	Glide score (Kcal/mol)
DB03213	ALA-141	ASP-161, GLU-208	-10.167
DB01705		GLU-208, ASP-161, CYX-22, GLY-20	-9.806
DB02559		GLU-208	-10.207
DB15199	ALA-141	ASP-161, GLY-23, SER-64, GLU-117, GLU-208, ASN-69	-9.253
DB00183	TRP-184, LEU-160	ASP-161, GLN-19, GLY-66,	-9.011
DB02704	CYS-25, TRP-184	ASP-161, SER-64, GLY-66, GLY-23, GLY-19	-11.177
DB03395	TRP-184	ASP-161, HIE-162, SER-64,	-10.856
DB04593	MET-68, LEU-160	GLU-208, ASN-69, GLY-66, GLN-19, GLY-23, ASP-161	-9.557

DB04869	LEU-160, TRP184	GLN-159, GLN-21, GLY-20,	-13.285
DB06763	LEU-160	GLY-66, ASP-161, ASP-140,	-9.596

Figure S1: The details of GPUs resource used in the experiment.

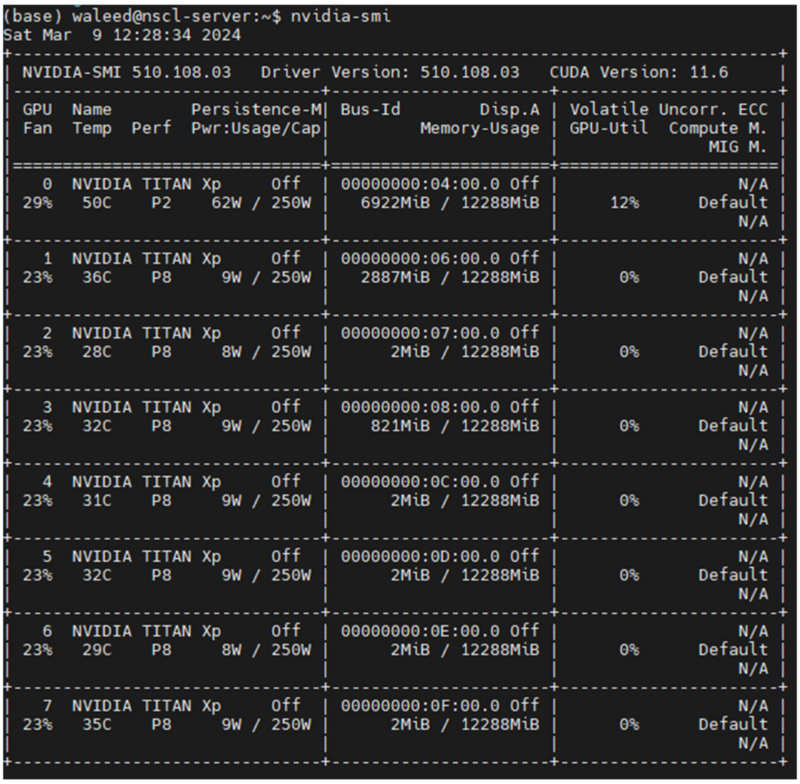

```
(base) waleed@nscl-server:~$ nvidia-smi
Sat Mar  9 12:28:34 2024
+-----------------------------------------------------------------------------+
| NVIDIA-SMI 510.108.03   Driver Version: 510.108.03   CUDA Version: 11.6     |
|-------------------------------+----------------------+----------------------+
| GPU  Name        Persistence-M| Bus-Id        Disp.A | Volatile Uncorr. ECC |
| Fan  Temp  Perf  Pwr:Usage/Cap|         Memory-Usage | GPU-Util  Compute M. |
|                               |                      |               MIG M. |
|===============================+======================+======================|
|   0  NVIDIA TITAN Xp     Off  | 00000000:04:00.0 Off |                  N/A |
| 29%   50C    P2    62W / 250W |   6922MiB / 12288MiB |     12%      Default |
|                               |                      |                  N/A |
+-------------------------------+----------------------+----------------------+
|   1  NVIDIA TITAN Xp     Off  | 00000000:06:00.0 Off |                  N/A |
| 23%   36C    P8     9W / 250W |   2887MiB / 12288MiB |      0%      Default |
|                               |                      |                  N/A |
+-------------------------------+----------------------+----------------------+
|   2  NVIDIA TITAN Xp     Off  | 00000000:07:00.0 Off |                  N/A |
| 23%   28C    P8     8W / 250W |      2MiB / 12288MiB |      0%      Default |
|                               |                      |                  N/A |
+-------------------------------+----------------------+----------------------+
|   3  NVIDIA TITAN Xp     Off  | 00000000:08:00.0 Off |                  N/A |
| 23%   32C    P8     9W / 250W |    821MiB / 12288MiB |      0%      Default |
|                               |                      |                  N/A |
+-------------------------------+----------------------+----------------------+
|   4  NVIDIA TITAN Xp     Off  | 00000000:0C:00.0 Off |                  N/A |
| 23%   31C    P8     9W / 250W |      2MiB / 12288MiB |      0%      Default |
|                               |                      |                  N/A |
+-------------------------------+----------------------+----------------------+
|   5  NVIDIA TITAN Xp     Off  | 00000000:0D:00.0 Off |                  N/A |
| 23%   32C    P8     9W / 250W |      2MiB / 12288MiB |      0%      Default |
|                               |                      |                  N/A |
+-------------------------------+----------------------+----------------------+
|   6  NVIDIA TITAN Xp     Off  | 00000000:0E:00.0 Off |                  N/A |
| 23%   29C    P8     8W / 250W |      2MiB / 12288MiB |      0%      Default |
|                               |                      |                  N/A |
+-------------------------------+----------------------+----------------------+
|   7  NVIDIA TITAN Xp     Off  | 00000000:0F:00.0 Off |                  N/A |
| 23%   35C    P8     9W / 250W |      2MiB / 12288MiB |      0%      Default |
|                               |                      |                  N/A |
+-------------------------------+----------------------+----------------------+
```
